# Evidence and Open Questions for the Use of Video-Feedback Interventions With Parents of Children With Neurodevelopmental Disabilities

**DOI:** 10.3389/fpsyg.2020.01374

**Published:** 2020-06-18

**Authors:** Livio Provenzi, Lorenzo Giusti, Marzia Caglia, Elisa Rosa, Eleonora Mascheroni, Rosario Montirosso

**Affiliations:** 0-3 Centre for the at-Risk Infant, Scientific Institute, IRCCS E. Medea, Lecco, Italy

**Keywords:** children, early intervention, parenting, rehabilitation, review, neurodevelopmental disabilities, video-feedback

## Abstract

The Video-Feedback Intervention (VFI) is a technique aimed at promoting positive parenting that has been found to be supportive of child development and parent–child interaction in different at-risk and clinical populations. The application of VFI with parents of children with neurodevelopmental disabilities (ND; e.g., cerebral palsy, sensory and/or psychomotor delay, and genetic syndromes) is growing. Nonetheless, no systematic review is currently available documenting whether this type of intervention improves children’s developmental outcomes (e.g., behavioral stability and cognitive abilities), parental caregiving skills (e.g., responsive parenting), and parental emotional well-being (e.g., depressive symptomatology). In the present mini-review, 212 VFI records were retrieved from three databases (i.e., PubMed, Scopus, and Web of Science), and 10 papers were finally included. Abstracted information included age, diagnosis, methodological aspects (timing, setting, and themes), and child/parent outcomes. Significant improvements from pre- to post-VFI were observed in all studies. Specifically, the VFIs were significantly associated with better children developmental outcomes and parental caregiving skills. Inconsistent findings emerged for the VFI effects on parental emotional well-being. Overall, the current mini-review supports the potential effectiveness of parent-focused VFI interventions for parents of children with ND, despite the presence of open questions that need to be addressed in future clinical trials.

## Introduction

In humans, parenting represents a key factor to promote the physical, socio-emotional, behavioral, and cognitive development of infants and children (Perrin et al., 2016; Provenzi et al., 2018). The role of parenting is much more relevant when infants and children present special healthcare needs due to neurodevelopmental risk or disabilities (Festante et al., 2019). Recent research shows that maximizing parental engagement and targeting parents’ caregiving skills alongside infants’ needs and disabilities are crucial for the success of early rehabilitation programs (Britto et al., 2017; Schuster and Fuentes-Afflick, 2017). Early parenting interventions are beneficial to improve developmental outcomes of infants and children and to limit some of the detrimental effects that special healthcare needs have on the quality of parent–child interaction (Spittle et al., 2015). In this article, we will review a specific approach to early parenting intervention (i.e., the video feedback intervention) and its application in children with special healthcare needs.

In 2016, about 53 million children worldwide received a diagnosis of neurodevelopmental disabilities (ND), representing 13% of all health problems in childhood (Olusanya et al., 2018). These children are a heterogeneous population with a variety of clinical diagnoses (e.g., cerebral palsy, sensory and/or psychomotor delay, genetic syndromes), which include several deficits that emerge very early in life (Ismail and Shapiro, 2019). Indeed, although diagnoses may vary, infants with ND partially share developmental impairments in physical (e.g., sensory deficits and motor development), emotion-behavioral (e.g., internalizing/externalizing problems), and cognitive domains (e.g., diminished attention span). As a consequence, children with ND can exhibit significant delay in two or more of the following developmental domains: gross/fine motor, speech/language, cognition, social/personal, and activities of daily living. A significant delay in two or more developmental domains affecting children under the age of 5 years is termed global developmental delay (Shevell et al., 2003). The presence of ND can have an impact on early interaction with caregivers, so that naturally occurring engagement processes are challenged and partially impeded (Spiker et al., 2002; Feniger-Schaal et al., 2019). For parents of these children, caregiving is much more complex than in typical development conditions (Giusti et al., 2018). First, parents face a significant emotional burden manifested as high levels of parenting stress, depressive and anxious symptoms (Findler et al., 2016). Second, the communicative signals of children with ND may be less clear for the parents to be interpreted and responded appropriately (Pennington and McConachie, 2001). In turn, less clear signals from the child might result in heightened parental intrusiveness, in the attempt to provide the child with regulatory and physical support (Azad et al., 2013). Moreover, these interactive and relational difficulties can ultimately increase the risk of developing behavioral problems in children (Spittle and Treyvaud, 2016). Importantly, several studies have documented that the quality of parenting is associated with children’s developmental outcomes, even in the presence of ND (Spiker et al., 2002; Assel et al., 2003; Festante et al., 2019). It has been shown that, beside social interaction and emotional support, parents also provide cognitive stimulation during their exchanges with their children, with long-term benefits for cognitive, language, and socio-emotional outcomes up to preschool- and school-age (Anderson et al., 2013; Innocenti et al., 2013; Totsika et al., 2019). Parental responsiveness and teaching associate with the developmental quotient of 23- to 47-month-old children with diverse ND (Vilaseca et al., 2019a). Notably, both paternal and maternal caregiving have been associated with better cognitive and language in development in preschoolers with ND (Vilaseca et al., 2019b). As such, early supportive interventions directed at improving the quality of parental caregiving and parent–infant interaction should be prioritized even in this population (Dyches et al., 2012; Spittle et al., 2015).

The Video Feedback Intervention (VFI) includes an array of procedures aimed at promoting positive parenting, which rely on theoretical principles of infant research tradition and have been used as stand-alone interventions or within extensive treatment programs at home or in hospital settings (Rusconi-Serpa et al., 2009; Groeneveld et al., 2011). VFI allows the parents to observe themselves “from the outside” as they interact with their own child. By promoting self-confrontation through video feedback review, the VFIs positively impact caregiving, with benefits for parental sensitivity and interactive attunement (Bakermans-Kranenburg et al., 2003). Different theoretical and methodological approaches to VFI are described in literature (e.g., Cohen and Beebe, 2002; Juffer et al., 2005, 2017; Schechter et al., 2006). Previous research has highlighted that VFI is associated with better child development and parent-child relationship in different clinical contexts, including children at risk for behavioral problems (Velderman et al., 2006; Balldin et al., 2018), preterm infants (Hoffenkamp et al., 2015; Barlow et al., 2016), hearing impairments (Santos and Brazorotto, 2018), maternal psychopathology (Rackett and Macdonald, 2014; Høivik et al., 2015; Kristensen et al., 2017), and ethnic minorities (Yagmur et al., 2014). Nonetheless, evidence on the effects of VFI in families of children with ND is sparse and lacks systematization. In light of this gap, this study aims (a) to describe the state of the art of VFI application in the presence of ND; (b) to synthesize VFI practice in this context on child and parent outcomes as well as on the quality of parent–child relationship; and (c) to highlight open questions for future research and reproducibility.

## Methods

### Literature Search

**FIGURE 1:**
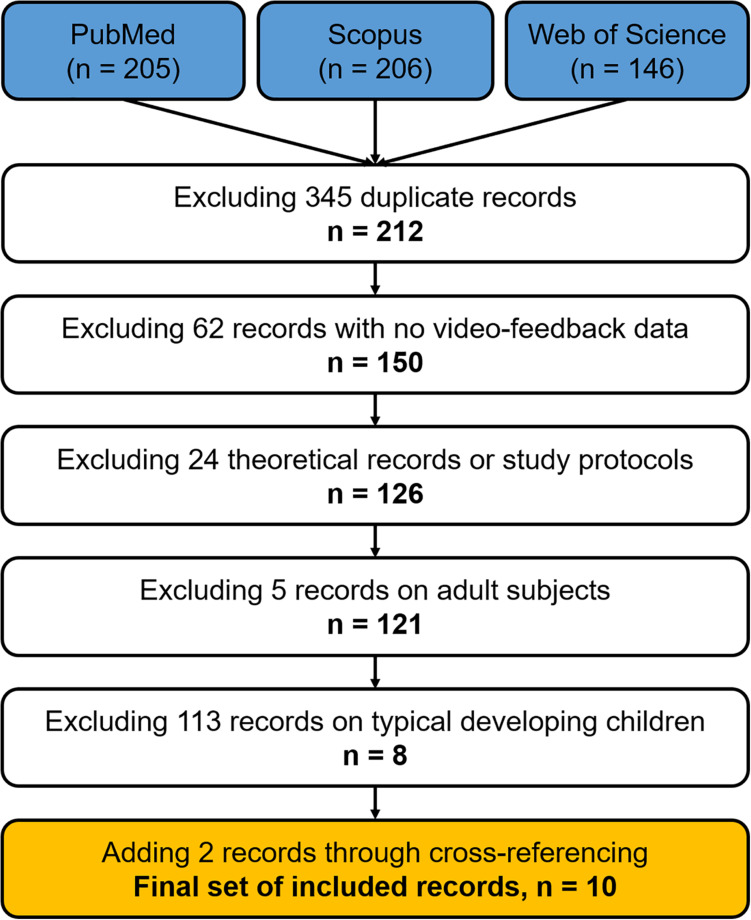
Flowchart of study selection.

The literature search was conducted on three databases (i.e., PubMed, Scopus, and Web of Science) with unconstrained time limits. A search string with an intentionally wide scope was used, with the following terms: (video feedback OR video-feedback) AND (infants OR children). The records were checked for duplicates using Endnote X5.01 (Thomson Reuters Scientific Inc., Carlsbad, CA, United States). The remaining papers were then filtered by two independent authors (i.e., ER and EM) by reading titles, abstracts, and the full articles. The presence of any neurodevelopmental risk (e.g., prematurity) or disability conditions with or without sensory impairment (e.g., hearing and visual) was checked through title/abstract screening as well as reading the full articles. Exclusion criteria were non-English language articles, animal studies, reviews, viewpoint papers, study protocols, absence of neurodevelopmental disability or sensorial deficits, and papers not focusing on parent–child relationship. Three additional records have been included through cross-referencing. The whole study selection process is reported in Figure 1.

The methodological quality of the included papers was assessed using the Quality Assessment Tool for Quantitative Studies (Jackson et al., 2005). Sections A–F (A, selection bias; B, study design; C, confounders; D, blinding; E, data collection methods; F, withdrawal and dropouts) were coded by two independent researchers (i.e., ER and EM) as 3 (weak), 2 (moderate), or 1 (strong) according to the component rating scale criteria. A final 1–3 score is assigned to each paper according to the presence of 2 or more weak scores (3, weak), only 1 weak score (2, moderate), no weak scores (1, strong). Ninety-six percent agreement was reached for the A–F components, and disagreement was generally due to different interpretations of studies. Disagreement was solved in conference by the supervision of the third author (RM). Quality appraisal is reported in Table 1.

**TABLE 1:** Quality appraisal of the included studies.

Study	A	B	C	D	E	F	Final
Mahoney and Powell (1988)	2	2	3	3	1	3	3
Seifer et al. (1991)	2	1	1	2	1	3	2
Kim and Mahoney (2005)	2	1	1	2	1	2	1
Phaneuf and McIntyre (2007)	3	1	3	3	3	2	3
Phaneuf and McIntyre (2011)	2	2	3	2	1	2	2
James et al. (2013)	3	2	3	2	1	3	3
Glanemann et al. (2013)	2	1	1	2	3	1	2
Lam-Cassettari et al. (2015)	2	2	1	2	1	1	1
Sealy and Glovinsky (2016)	2	2	1	2	1	1	1
Platje et al. (2018)	2	1	1	2	1	1	1

*Labels: A, selection bias; B, study design; C, confounders; D, blinding; E, data collection methods; F, withdrawals and dropout. Quality codes: 1, strong; 2, moderate; 3 weak.*

### Data Abstracting

A final pool of 10 studies was selected (Table 2). The records were reviewed, and the following data were extracted: authors, year of publication, journal, children characteristics, maternal characteristics, age at start, procedure, setting, number of sessions, frequency of sessions, outcome variable(s), and findings. Data were analyzed according to the aims of the present review. We decided to abstract information about infants’ characteristics related to the ND condition because previous research suggest that parents’ well-being AND/OR parent–infant interaction is affected by the severity of infants’ clinical condition (Smith et al., 2001). Similarly, the quality of parenting and the effect of parental caregiving may also vary as a function of infants’ age (Woolfson and Grant, 2006); as such, we abstracted the age of infants at the start of the included VF interventions. Setting was also abstracted, because parent–children interaction may be different at home or in lab environments (McWilliam et al., 2000), and it would be important for us to document if these VF interventions have been provided at home or in hospitals/rehabilitation centres. Finally, the number and frequency of sessions have been abstracted to control for the different effect of these procedural characteristics on the outcomes.

**TABLE 2:** Characteristics of the studies included in the review.

Study	Study design	VFI protocol	Setting	Sample size (dyads)	Sessions number	Sessions frequency	Children age	Clinical category
Mahoney and Powell (1988)	Trial	TRIP	Home	41	N.A.	>Weekly	2–32 months	ND
Seifer et al. (1991)	Trial	N.A.	Hospital	40	6	Weekly	8.5 months	ND
Kim and Mahoney (2005)	Trial	RFI	Home	18	2	Weekly	3–8 years	ND
Phaneuf and McIntyre (2007)	Case study	IVF	Home	4	1–3	>Weekly	2–4 years	ND
Phaneuf and McIntyre (2011)	Case study	N.A.	Home	8	1–3	N.A.	2–4 years	ND
James et al. (2013)	Case study	VIG	Home	3	3	>Weekly	9–36 months	Hearing impairments* and ND
Glanemann et al. (2013)	Trial	MPP	Hospital	29	8	Weekly	3–6 months	Hearing impairments* and ND
Lam-Cassettari et al. (2015)	Trial	N.A.	Hospital	14	3	N.A.	2.5 years	Hearing impairments* and ND
Sealy and Glovinsky (2016)	Trial	DIR/FT	Hospital	40	12	N.A.	2–6 years	ND
Platje et al. (2018)	Trial	VIPP-V	Home	40	7	>Weekly	1–5 years	Visual or visual-and-intellectual disability

N.A., not available; ND, neurodevelopmental disability; TRIP, Transactional Intervention Program; RFI, Relationship-Focused Intervention; IVF, Individualized Video Feedback; VIG, Video Intervention Guidance; DIR/FT, Developmental Individual-difference, Relationship-based/Floortime Intervention; VIPP-V, Video feedback Intervention to promote Positive Parenting in parents of children with visual or visual-and-intellectual disabilities; MPP, Muenster Parental Program; *, onset of hearing impairments is prelingual.

### Data Synthesis

First, (a) an in-depth description of different VFI approaches and methodologies is reported, including theoretical underpinnings, techniques, procedures, setting, and timing. Second, (b) effects of VFI on child outcomes, parental well-being, and the quality of parent–child interaction were reviewed. Finally, (c) inconsistencies in methodology were highlighted to inform future research advances and clinical practice.

Data synthesis occurred according to the following clusters: (1) children characteristics, (2) VFI methodology (i.e., procedures, setting, and sessions), and (3) outcomes for child development, parental well-being, and parent–child interaction.

## Findings

### VFI State of the Art and Methodology

#### Characteristics of Participating Subjects

The included studies focused on different ND, including cerebral palsy, genetic syndromes with psychomotor delay or non-specified developmental delay (Mahoney and Powell, 1988; Seifer et al., 1991; Kim and Mahoney, 2005; Phaneuf and McIntyre, 2007, 2011; Sealy and Glovinsky, 2016), visual disability (Platje et al., 2018), and hearing problems (Glanemann et al., 2013; James et al., 2013; Lam-Cassettari et al., 2015). Children’s age widely varied among the included studies: the VFI was delivered during the first years of life (from 2 to 36 months) in four studies (Mahoney and Powell, 1988; Seifer et al., 1991; Glanemann et al., 2013; James et al., 2013), during preschool age in five studies (Phaneuf and McIntyre, 2007, 2011; Lam-Cassettari et al., 2015; Sealy and Glovinsky, 2016; Platje et al., 2018), and up to 8 years of age in a mixed sample of preschool and school-aged children (Kim and Mahoney, 2005). A schematic overview of the findings from the original records is included in Supplementary Table S1.

#### VFI Approaches: Procedures and Methodology

Video-Feedback Intervention protocols varied in terms of setting, main target themes, and timing of sessions. The Transactional Intervention Program (TRIP) is an early home-based intervention for parents of 0- to 3-year-old children to promote responsive parenting, by encouraging parents to adopt specific strategies in their daily interactions with their child (Mahoney and Powell, 1988). Main themes include turn-taking and interactive matching. The TRIP video feedback is applied every 6–10 weeks. The Relationship-Focused Intervention (RFI) (Kim and Mahoney, 2005) is a home-based 3-month-long intervention made up of four components: classroom-based instruction, home-based instruction, video feedback, and evaluation. The video feedback is implemented for two sessions. As for the TRIP, turn-taking and interactive matching strategies are the main target themes. The home-based Individualized Video Feedback (IVF) consists of a 3-session program over a 6-week period, providing feedback to the parents on the strengths and weaknesses of their interactive behaviors (Phaneuf and McIntyre, 2007). The Video Interaction Guidance (VIG) is a 3-session intervention aimed at facilitating the establishment of parental feelings of bonding toward the infant after birth. The VIG may be applied at home- and in hospital settings (James et al., 2013; Lam-Cassettari et al., 2015). The VIG standardized protocol (i.e., video-recording, editing, and reviewing edited recordings with parents) includes preliminary sessions in which therapist and parents co-define the intervention’s goals. A three-tier model of intervention is used by Phaneuf and McIntyre (2011) that consists in self-administered reading material, group training, and individualized video feedback sessions based on strengths and weaknesses of the parents and children behavior. The Developmental Individual-difference Relationship (DIR) focuses on parental attunement to child’s sensory processing abilities (i.e., the way each child takes in, regulates, responds to, and understands sensory stimulations) in order to reinforce co-regulation processes and to reduce disruptive interactive sequences (Sealy and Glovinsky, 2016). Free play interactions between the parent and the child are video-recorded in the hospital setting for subsequent dialogic sessions with the therapist. The number of sessions is not fixed. The Video feedback Intervention to promote Positive Parenting in parents of children with Visual or Visual-and-intellectual disabilities (VIPP-V) (Platje et al., 2018) is a home-based program adapted from the original VIPP from Juffer et al. (2005). Up to seven sessions with varying time intervals focus on specific predetermined themes including exploration versus attachment behavior, speaking for the child, sensitive interactive exchanges, and sharing emotions. An additional focus of interest includes quality of interaction, intersubjectivity, and joint attention. The Muenster Parental Program (MMP) was developed to enhance responsive parental behavior to the child’s vocal and non-verbal signals, and to reduce parental behavioral intrusiveness (Glanemann et al., 2013). The MMP is composed of six group sessions and two individual training sessions, and it focuses on the following themes: waiting for the child’s initiation, following the child’s interest, mirroring vocal and preverbal signals, mirroring the child’s non-verbal signals (movements and actions), and offering expanding feedback. Finally, Seifer et al. (1991) used a hospital-based VFI coaching program that lasted for six weekly sessions and focused on dimensions of reciprocal interaction, non-contingency, and overstimulation.

### Impact of the VFI in Neurodevelopmental Disability

#### Effects on Child Behavior and Developmental Outcomes

Significant reduction of aggressive, disruptive, and emotionally negative behaviors was reported by Phaneuf and McIntyre (2011) in 2- to 4-year-old children with ND. In children with hearing impairment, behavioral problems were found to significantly decrease at the post-intervention assessment with long-lasting effects up to the 3-month follow-up (James et al., 2013). Increased communicative skills and higher developmental quotient were reported by Seifer et al. (1991) in a sample of children with ND. Also Glanemann et al. (2013) found an increase in vocalization behavior in 3- to 18-month children with hearing loss whose parents had participated in the training. James et al. (2013) showed that children with moderate-to-severe ND (i.e., Down syndrome, undetermined cognitive impairment) whose parents attended the VFI increased vocal autonomy, communicative, and vocal productions and were more able to actively interact with the caregiver. Moreover, after the intervention, children with prelingual deaf and hard of hearing showed a better interactive behavior in terms of involvement and responsivity with parents (Lam-Cassettari et al., 2015). Finally, in 2- to 32-month ND children, a higher developmental quotient in association with VFI was also documented by Mahoney and Powell (1988).

#### Effects on Parent–Child Relationship and Parental Interactive Behavior

The majority of the studies were aimed at modifying maternal behavior in the context of mother–child interaction. Nonetheless, different dimensions of maternal caregiving have been targeted by the diverse VFI approaches, such as interactive turn-taking and matching, contingency and responsiveness, amount of stimulation and intrusiveness, affective behavior, scaffolding of verbal communications and attention, and reduction of inappropriate behaviors. The VFI has been found beneficial to promote better turn-taking strategies, higher matching, better reciprocity and higher responsivity (Mahoney and Powell, 1988; Seifer et al., 1991; Glanemann et al., 2013; Sealy and Glovinsky, 2016), the capacity to promptly and contingently respond to the child’s communicative bids (Kim and Mahoney, 2005; James et al., 2013), the adoption of affective behaviors as well as positive strategies to support child behavioral stability (James et al., 2013; Phaneuf and McIntyre, 2011), and the ability to give meaning to children’s behaviors (i.e., reflective functioning; Sealy and Glovinsky, 2016). Moreover, a reduction in the amount of stimulation and intrusiveness (Mahoney and Powell, 1988; Seifer et al., 1991; Glanemann et al., 2013) as well as in the adoption of inappropriate (Phaneuf and McIntyre, 2007, 2011) and hostile (Lam-Cassettari et al., 2015) behaviors was also observed.

#### Effects on Parental Well-Being

The impact of VFI on parental psychological health has received far less attention. Improved maternal well-being has been assessed and considered as a reduction in at least one of the following domains: parenting stress (Kim and Mahoney, 2005; Platje et al., 2018), capacity to develop an intimate bond with the child, feeling of enjoyment in the interaction with the child and self-esteem (Lam-Cassettari et al., 2015), and parental self-efficacy (Platje et al., 2018). An inconsistent pattern of results emerged. While two studies found a reduction in parenting-related stress in families of children with psychomotor delay (Kim and Mahoney, 2005; Platje et al., 2018), no significant improvement has been documented in families of congenitally deaf and preverbal children (Lam-Cassettari et al., 2015).

## Discussion

The present mini review was aimed at summarizing the evidence on the application of VFIs with parents of children with ND. The promotion of positive parenting and relational interventions is more and more advocated in the field of ND, as they have the highest probability of resulting in long-lasting protective effects on child development and family well-being (Spittle and Treyvaud, 2016). Moreover, it has been demonstrated that parenting interventions that start before preschool age are the most effective, as they appear to be associated with greater economical return for healthcare systems (Doyle et al., 2009). Notably, despite VFIs have been used successfully with different at-risk children populations (Hoffenkamp et al., 2015), only 10 records were retrieved, suggesting that, currently, the application of video feedback to the population of ND children is only partially documented in scientific literature.

### VFI With Infants Affected by Neurodevelopmental Disability and Their Parents: A Promising Supportive Intervention

All the studies included reported positive outcomes of VFI on ND children and their parents. First, positive effects on children’s development emerged, including reduced behavioral problems, improved cognitive outcomes, and interpersonal functioning. Second, parents showed increased capacity to read and respond to children’s signals, and there was a consistent positive effect on the quality of parent–infant interaction in terms of reciprocity and mutual regulation. Notably, these effects were documented in all the studies, independently of children diagnosis, degree of impairment, and age. Such cross-disability effect speaks in favor of considering VFI strategies as optimal early interventions that may be pursued both in hospital settings and in the family home environment. Notably, limited evidence on the improvement of maternal well-being and emotional adjustment emerged. Whereas only a limited subset of studies (*n* = 3) investigated the effects of video feedback methodology on parental stress, depression, and/or anxiety, it should be noted that the main VFI target focused on parental skills and infant/child behavior. Improving children behavior (i.e., emotion regulation) and parental skills might be beneficial to reduce parenting stress in some cases. Nonetheless, the reduction of depressive and anxious symptomatology in parents may only be partially achieved through interaction-focused interventions such as the VFI, especially when parents are facing the chronic and highly demanding ND conditions of their child. Consistently, the lack of a direct effect in promoting parental psychological health suggests that the VFI should be integrated with other parent-directed interventions when concerns for parental psychological health are present.

### Open Questions for Clinically Relevant Research

The above-presented findings generate several open questions that highlight the need of further evidence-based clinical practice in children with ND. First, from a methodological point of view, according to previous review on VFI in at-risk children (Balldin et al., 2018), in the included studies emerged a couple of critical issues: low specificity of programs with respect to the VFI features and heterogeneity among measures used for assessments. Thus, a major goal of future research might be the promotion of international consortia of clinicians involved in VFI applied research with ND children. Second, only four out of 10 records obtained the highest quality appraisal score. This appears to be related, at least partially, to the fact that many papers reporting on the effect of VFI with parents of ND children were single case studies. To increase the generalizability and reliability of findings, future research should be directed at testing the effect of VFI in properly designed randomized or quasi-randomized clinical trials. Third, there is still a lack of studies assessing the effects of VFI involving fathers, rather than only mothers. Fathers represent a crucial component of infants’ primary care, especially in ND populations (Provenzi et al., 2016; Fisher et al., 2018). As such, the study of VFI impact with fathers and/or engaging both parents simultaneously is highly warranted. Fourth, the effects of VFI on both parenting skills and children development are generally cross-disability, which is also suggestive of the possibility to conduct studies on the effects of such early interventions on children with specific NDs (e.g., Down syndrome). However, selecting children based on specific diagnosis might result in very limited sample size and under-powered studies. Therefore, the present review suggests that future studies may avoid using diagnosis-specific criteria for defining the parent–child population included in VFI trials, in order to have adequately powered study designs while maximizing the translational value. Finally, it should be highlighted that studies reporting on the effects of either stand-alone video feedback or parenting programs in which video feedback was part of a broader intervention were included in this review. As such, it was not possible to investigate the specific benefits of VFI when it was embedded in more complex and integrated intervention programs. Nonetheless, from a clinical perspective, the integration of different intervention methods constitutes an optimal strategy to respond to the multi-faceted needs of children with ND and their parents, especially in the presence of multiple risk situations and major clinical-care needs.

## Conclusion

Promoting infants and children’s development through the active engagement of parents should be a priority in the presence of children with ND (Guralnick, 2005; Schuster and Fuentes-Afflick, 2017). Family centred interventions directed at the parent–infant system should be promoted during the early stages of infants’ development (Schuster and Fuentes-Afflick, 2017) in order to maximize their efficacy and to be beneficial for both families and the healthcare systems (Doyle et al., 2009). The VFI appears to be a very promising and effective approach. The present review suggests that specific parental behaviors (e.g., sensitivity and contingent caregiving) and interactive features (e.g., promotion of turn-taking and joint attention) can greatly benefit from VFI programs. Nonetheless, future research should be directed at testing the effectiveness of VFI through appropriately designed randomized clinical trials. Moreover, the VFI should not be used in a one-size-fits-all approach and should be implemented carefully both in home- and hospital-based settings. The clinician’s specific knowledge of typical and atypical development as well as of mother–infant interaction is crucial, which means that VFI should be applied and delivered only by well-trained healthcare professionals with an adequate background and experience in the field. Finally, the integration of VFI protocols with validated individual interventions directed at promoting either psycho-motor adjustment of children and parental emotional well-being should be pursued in clinical settings and adequately documented in future studies.

## Author Contributions

LP and LG conceived the study and wrote the first draft of the manuscript. EM, MC and ER were responsible for data collection and analysis. RM provided methodological supervision, reviewed and edited the writing. All authors approved the final version of the manuscript.

## Conflict of Interest

The authors declare that the research was conducted in the absence of any commercial or financial relationships that could be construed as a potential conflict of interest.
