# Supplementary material for: Evidence and Open Questions for the Use of Video-Feedback Interventions With Parents of Children With Neurodevelopmental Disabilities
Source: Front Psychol. 2020 Jun 18;11:1374. doi: 10.3389/fpsyg.2020.01374 (PMC7314919; doi:10.3389/fpsyg.2020.01374)
Supplement: Supplementary file 1 [file Table_1.DOCX]

**Supplementary table S1.** Statistics details for the included studies and the main effects reported.

1. Effects on child outcomes

| **Study** | **Outcome measure** | **Study design** | **Direction of effect** | **Mean 1 (SD)** | **Mean 2 (SD)** | **Statistic test** | **Effect size** |
| --- | --- | --- | --- | --- | --- | --- | --- |
| Mahoney & Powell (1988) | Overall developmental gain | Within-group | 🡹 | N/A | N/A | *F* = 2.84 * | R^2^ = .33 |
| Seifer et al. (1991) | Overall developmental gain | Between-group | 🡹 | 0.21 (0.83) | -0.50 (1.20) | *t* = 2.15 * | d = 0.69 |
| Phaneuf & McIntyre (2011) | Inappropriate behaviors | Within-group | 🡻 | 8.33 (8.17) | 0.42 (1.18) | *t* = 2.89 * | d = 0.15 |
| Glanemann et al. (2013) | Vocalizations | Between-group | 🡹 | 16.5 (1.9) | 9.6 (1.3) | *t* = 2.99 ** | d = 4.23 |
| Lam-Cassettari et al. (2015) | Child involvement | Within-group | 🡹 | 4.79 (NA) | 5.38 (NA) | *F* = 41.35 ** | η^2^_p_ = .78 |

Note. The following papers did not report statistics for child outcomes: Kim & Mahoney, 2005, Phaneuf & McIntyre, 2007, James et al., 2013, Sealy & Glovinsky, 2016, Platje et al., 2018. For within-group study design: Mean 1, Pre-intervention; Mean 2, Post-intervention. For between-group study design: Mean 1, Intervention group; Mean 2, Control group. **p* value <.05; ***p* value <.01. Effect size was included in these papers as reported in the original papers. When effect size was not included in the original papers, it was estimated as Cohen’s *d* effect size index using formulas from available data (e.g., *d* = t/RADQ(N); *d* = Mean_difference_ / S_pooled_).

1. Effects on maternal behavior

| **Study** | **Outcome measure** | **Study design** | **Direction of effect** | **Mean 1 (SD)** | **Mean 2 (SD)** | **Statistic test** | **Effect size** |
| --- | --- | --- | --- | --- | --- | --- | --- |
| Kim & Mahoney (2005) | Parental stress | Between-group | 🡻 | 21.0 (7.60) | 27.50 (2.98) | *F* = 8.44** | ES = 0.35 |
| Lam-Cassettari et al. (2015) | Self-esteem | Within-group | 🡹 | 20.42 (NA) | 22.71 (NA) | *F* = 4.81* | η^2^_p_ = .29 |
| Platje et al. (2018) | Parenting self-efficacy | Between-group | 🡹 | 93.61 (12.48) | 91.00 (10.30) | *F* = 5.35* | η^2^ = .07 |

Note. The following papers did not report statistics for parent outcomes: Mohoney & Powell, 1988; Seifer et al., 1991; Phaneuf & McIntyre, 2007, 2011; James et al., 2013; Glanemann et al., 2013; Sealy & Glovinsky, 2016. For within-group study design: Mean 1, Pre-intervention; Mean 2, Post-intervention. For between-group study design: Mean 1, Intervention group; Mean 2, Control group. ES, non-specified effect size estimation index. *, p value <.05; **, p value <.01. Effect size was included in these papers as reported in the original papers. When effect size was not included in the original papers, it was estimated as Cohen’s *d* effect size index using formulas from available data (e.g., *d* = t/RADQ(N); *d* = Mean_difference_ / S_pooled_).

1. Effects on interactive behaviors

| **Study** | **Outcome measure** | **Study design** | **Direction of effect** | **Mean 1 (SD)** | **Mean 2 (SD)** | **Statistic test** | **Effect size** |
| --- | --- | --- | --- | --- | --- | --- | --- |
| Mahoney & Powell (1988) | Responsiveness | Within-group | 🡹 | 3.09 (1.20) | 3.75 (1.24) | *t* = 2.53* | d = 0.40 |
| Mahoney & Powell (1988) | Directiveness | Within-group | 🡻 | 3.58 (1.22) | 2.80 (1.15) | *t* = 3.28** | d = 0.51 |
| Seifer et al. (1991) | Responsiveness | Between-group | 🡹 | .23 (.57) | -.31 (.55) | *t* = 1.76* | d = 0.29 |
| Seifer et al. (1991) | Stimulation | Between-group | 🡻 | -.25 (.93) | .35 (.83) | *t* = 2.02* | d = 0.32 |
| Kim & Mahoney (2005) | Responsiveness | Between-group | 🡹 | 3.20 (0.73) | 1.77 (0.53) | *F* = 16.19*** | ES = 0.50 |
| Glanemann et al. (2013) | Responsiveness | Between-group | 🡹 | .50 (.07) | .20 (.05) | *t* = 3.17** | d = .65 |
| Glanemann et al. (2013) | Intrusiveness | Between-group | 🡻 | 3.7 (1.2) | 26.0 (3.1) | *t* = -6.74** | d = 1.38 |
| Lam-Cassettari et al. (2015) | Hostility | Between-group | 🡻 | 6.57 .45) | 5.96 (.98) | *F* = 6.88* | η^2^_p_ = .36 |
| Sealy & Glovinsky (2016) | Reflective functioning | Between-group | 🡹 | 5.35 (.28) | 4.15 (.28) | *F* = 13.39** | η^2^ = .62 |

Note. The following papers did not report statistics for interaction behavior outcomes: Phaneuf & McIntyre, 2007, 2011; James et al., 2013; Platje et al., 2018. For within-group study design: Mean 1, Pre-intervention; Mean 2, Post-intervention. For between-group study design: Mean 1, Intervention group; Mean 2, Control group. **p* value <.05; ***p* value <.01; ****p* value <.001. Effect size was included in these papers as reported in the original papers. When effect size was not included in the original papers, it was estimated as Cohen’s *d* effect size index using formulas from available data (e.g., *d* = t/RADQ(N); *d* = Mean_difference_ / S_pooled_).
